# Development of a Gas Chromatography-Mass Spectrometry Method for the Quantification of Glucaric Acid Derivatives in Beverage Substrates

**DOI:** 10.1155/2014/402938

**Published:** 2014-06-15

**Authors:** Ana Paula Craig, Christine C. Fields, John V. Simpson

**Affiliations:** ^1^Applied Food Sciences, Inc., 8708 South Congress, Austin, TX 78745, USA; ^2^Chemir Analytical Services, 2672 Metro Boulevard, Maryland Heights, MO 63043, USA

## Abstract

A gas chromatography-mass spectrometry (GC-MS) method using the standard addition methodology was developed for the determination of glucuronolactone (GL) and glucuronic acid (DGuA) in four beverages categorized as detoxification, recovery, or energy drinks. The method features a precolumn derivatization step with a combination of BSTFA (N,O-bis(trimethylsilyl)trifluoroacetamide) and TMCS (trimethylchlorosilane) to silylate the analytes. The sample pretreatment required no extraction, filtration, or reduction step prior to the injection. The quantification of the analytes was performed using a five-point standard addition protocol. The proposed method presented excellent intraday precision (%RSD < 10) and linearity for GL calibration curves (correlation coefficients > 0.995) and acceptable linearity for DGuA calibration curves (correlation coefficients > 0.97). The estimated limits of detection (LOD) and quantification (LOQ) for GL ranged from 0.006 ppm to 0.14 ppm, and 0.02 ppm to 0.47 ppm, respectively. The estimated LOD and LOQ for DGuA determination ranged, respectively, from 0.06 ppm to 1.1 ppm and 0.2 ppm to 3.8 ppm. The results demonstrated that the method should be regarded as a reliable alternative to the simultaneous determination of GL and DGuA.

## 1. Introduction

D-glucaric acid (DGA) and its derivatives, DGuA and GL, besides being naturally found in fruits and vegetables and being endogenously produced in mammals [1], are available in different drug formulations and beverages. These compounds have been associated with several therapeutic uses, including cholesterol reduction [1], protective effects against oxidative/nitrative damage of human plasma proteins [2], diabetes treatment [3], and cancer prevention [4–6]. In combination with other constituents of the so-called energy drinks, such as caffeine, taurine, and B-vitamins, these compounds claim to enhance physical endurance and reduce fatigue [7].

Different methods have been reported in the literature with the purpose of quantifying DGA. The former methods are enzymatic assays modified from the method proposed by Marsh [8] and measure the GL inhibition of *β*-glucuronidase. As this inhibition occurs in the presence of GL, the sample is boiled at an acidic pH to establish equilibrium between DGA and GL. The results from this assay, however, may lead to errors if different factors such as boiling, pH, and time are not rigorously kept constant [9]. The determination of DGA and its derivatives in urine samples after acidification was attempted by Gangolli et al. [10] and Laakso et al. [11], using gas chromatography (GC) and high-performance liquid chromatography (HPLC) methods, respectively. In the method proposed by Laakso et al. [11], the results were only qualitative, as the GLs peaks did not separate well from the DGA peak. In the study by Walters et al. [12], a HPLC method for the detection of DGA and other metabolites in urine by radioactivity monitoring and UV absorption was described. Although the DGA peak could be resolved and separated from other compounds, a quantitative assay was not attempted. Walaszek et al. [1] employed a pyruvate assay to quantify, nonselectively, DGA, its salts, and its lactones, in various fruits and juices. In this method* Escherichia coli* catabolic enzymes convert DGA and its derivatives to pyruvate, which is then analyzed via lactate dehydrogenase. More recently, Perez and coworkers [13] quantified the content of DGA in grapefruits by HPLC using a simple isocratic mobile phase and a versatile sample preparation that involved only homogenization, centrifugation, and filtration through a 0.45 *μ*m membrane.

Although many studies have reported the analysis of DGA in different matrices, the quantification of the DGA derivatives, for example, GL and DGuA, has not been extensively explored. Honda et al. [14] proposed a method based on high-performance anion-exchange chromatography to simultaneously determine uronic acids including DGuA in some polyuronides. Although the results were consistent on a qualitative basis, the quantification failed to yield the expected amount of uronic acids due to the incomplete release and partial degradation during acid hydrolysis. Tisza et al. [15] reported that the TMS oxime derivatives of sugar acids, neutral sugars, and other organic acids from different apple varieties could be simultaneously analyzed by GC-MS. The determination of the components was based on the evaluation of their total ion count data applying external standards and in selected ion monitoring. Nevertheless, for the identification and determination of those compounds that were not available in the standard solution, such as DGA, gluconic acid, and DGuA, the closest eluting member of the standard solution served as the basis for the calculation. Suzuki et al. [16] reported a methodology to quantify DGuA and GL in drug formulations and beverages using HPLC and a UV spectromonitor working at 245 nm. The method involved a precolumn derivatization to hydrolyze the intraester linkage of GL to its free-acid form, yielding DGuA derivatives, exclusively. More recently, Matsumoto et al. [17] reported a sensitive HPLC method with fluorometric detection and postcolumn derivatization for the determination of uronates, including DGuA, isolated from polysaccharides found in natural products such as alginate.

In view of the aforementioned, the objective of this work was to develop and validate a reliable analytical methodology based on GC-MS for the determination of DGA derivatives at low levels in commercial beverages. To our knowledge, this is the first time that a GC-MS method is reported to simultaneously quantify GL and DGuA in beverages.

## 2. Experimental

### 2.1. Reagents and Materials

The GL and DGuA standards were supplied, respectively, from Anhui Fubore Pharmaceutical & Chemical (China) and Sigma-Aldrich (USA). DGA was supplied from Applied Food Sciences, Inc. Stock standards of each compound were prepared by weighing and diluting into a vial 0.05 g of the standard to 30 g with dimethilformamide (DMF) (Sigma-Aldrich, USA). The stock standards were sonicated to achieve complete dissolution. The mixed stock standard was prepared by weighing and mixing into a vial 1.5 g of the GL stock standard and 4.5 g of the DGuA stock standard and diluting the mixture to 25 g with DMF. The calibration stock standards were prepared by taking aliquots of the mixed stock standard and diluting to mass with DMF, at seven different levels, as shown in Table 1. Dichloromethane (DCM) obtained from Sigma-Aldrich (USA) was used as solvent, and BSTFA + 1% TMCS purchased from Sigma-Aldrich (USA) was used as a silylation reagent. Furthermore, a system suitability standard and a blank were prepared to investigate the method qualification. The system suitability standard was obtained by mixing 0.5 g of the calibration stock at level 5, 0.5 g of DMF, and 0.5 mL of BSTFA + 1% TMCS, sonicating for complete dissolution, diluting to 2.5 g with DCM, and centrifuging the solution. The blank was prepared by mixing 0.5 g of DMF and 0.5 mL of BSTFA + 1% TMCS, sonicating the solution, diluting to 2.5 g with DCM, and centrifuging.

### 2.2. Sample Preparation

Four different beverages categorized as recovery or energy drinks were purchased in local supermarkets. Beverages codified as 1 and 2 have DGA as the active ingredient. The presence of GL and DGuA in these beverages is expected, as under acidic and neutral conditions DGA is in equilibrium with its intramolecular esters and lactones [18]. Beverages 3 and 4, besides containing stimulant ingredients such as caffeine, contain GL as an active ingredient. The samples were stored at room temperature. Samples 2, 3, and 4 were transferred into a preweighted round bottom flask and lyophilized to residue. The samples were frozen with liquid nitrogen and then placed on the lyophilizer, with a pressure of 0.180 mbar and −51°C, until water was completely removed. It was not necessary to lyophilize sample 1 as it was already in powder form.

**Figure 1:**
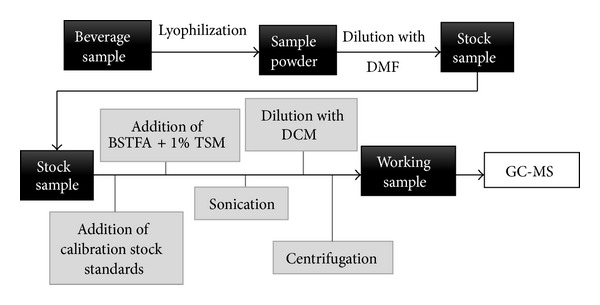
Schematic of working sample preparation.

The stock samples were prepared in duplicate weighing aliquots of the lyophilized sample into a vial, diluting to mass with DMF, and sonicating to achieve complete dissolution. The approximate dilution ratio, in grams, for sample 1 was 0.5 : 5, for sample 2, 0.25 : 7.5, and, for samples 3 and 4, 0.25 : 5. For the preparation of the working samples, five aliquots of the stock samples were weighted into 8 mL vials. The mass of aliquots for samples (a) 1, (b) 2, and (c) 3 and 4 was, respectively, 0.25 g, 0.75 g, and 0.5 g. Then, 0.25 g of the calibration stock standards at five different standard levels was added to each vial. The standard levels added to samples 1 and 2 were L1, L2, L3, L4, and L5, and the levels added to samples 3 and 4 were L1, L4, L5, L6, and L7. Sequentially, 0.5 mL of BSTFA + 1% TMCS was added to each vial. The vials were sealed and sonicated for five minutes. The samples were diluted to 2.5 g with DCM and centrifuged. A schematic of the sample preparation is shown in Figure 1.

### 2.3. GC-MS Equipment and Conditions

A gas chromatograph 6890 (Agilent, USA) equipped with an electronically controlled splitless injection port and coupled with a single quadrupole inert mass selective detector (5973, Agilent, USA) with electron impact ionization chamber was used for the GC-MS analysis. GC separation was performed on a DB-624 capillary column (60 m × 0.25 mm × 1.4 *μ*m) (J&W Scientific, USA). Helium was the carrier gas with a constant pressure of 25.6 psi. About 1 *μ*L of the sample solution was injected in splitless mode at 260°C. The initial temperature of the oven was 150°C and ramped with a rate of 10°C per minute until achieving 260°C. The temperature was held at 260°C for not less than 25 minutes. Mass spectrometric parameters were set with electron impact ionization energy of 69.9 eV, ion source temperature of 230°C, and MS quadrupole temperature of 150°C. The MS system was routinely set in selective ion monitoring (SIM) mode. The target peaks assignments were confirmed with genuine materials. GL was quantified based on peak area using the extracted ion 230, while DGuA was quantified based on the extracted ion 217.

### 2.4. Standard Addition Method

**Figure 2:**
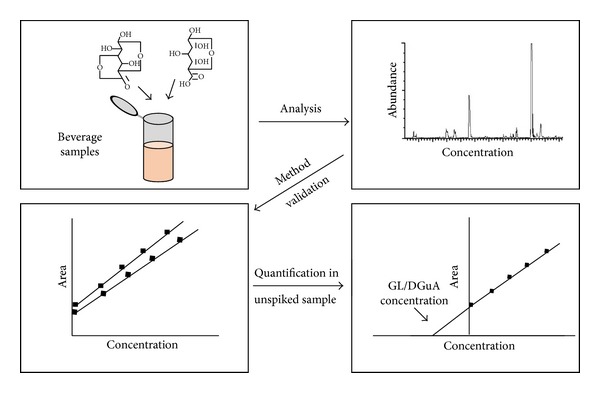
Schematic of GC-MS method using the standard addition method to quantify GL and DGuA in beverages.

The quantification of the analytes was performed in duplicate by the method of standard addition as follows. For each beverage sample, a calibration curve for GL and another for DGuA were developed by linear regression models. Each calibration curve was constructed from sets of five GC peak areas of the analyte (one from the unspiked working sample and four from the spiked working samples with different levels of standard solution). The peak area values at each addition were plotted as *y*-axis* versus* the concentrations of GL and DGuA spiked in the working samples, which were plotted as the *x*-axis. The analyte concentration in the unspiked working samples was determined by extrapolating the calibration curve to the negative part of the concentration axis [19]. Then, the absolute value of the* x*-intercept was calculated as the ratio between the* y*-intercept and the slope. As the analysis involved a derivatization and a standard addition curve at low concentrations, the acceptance criteria for the calibration curve were determined as a correlation coefficient (*R*²) greater than 0.97. The absolute value for the *x*-axis obtained from the calibration curve, when the value of the *y*-axis was equal to zero, was calculated as the amount of GL and DGuA in the unspiked working sample, which represents the actual content of these compounds in the beverage samples under study.  Figure 2 demonstrates the schematic of the standard addition methodology.

## 3. Results and Discussion

### 3.1. Optimization of Method Conditions

An initial feasibility work was performed to establish the ideal extraction conditions of the analytes. BSTFA + 1% TMCS was chosen to silylate the analytes, and DMF was the solvent of choice because of its polar nature. In order to establish the identity of the target analytes, a mix standard containing approximately 50 ppm of DGA, GL, and DGuA was prepared. Although DGA was not soluble in DMF, its derivatized form (DGA-TMS) was. At this initial attempt, a 30 meter DB-5MS capillary column (J&W Scientific, Folsom, CA, USA) was used for the GC analysis. The standard mix was analyzed and the derivatized analytes could be clearly identified. However, when a portion of sample 1 was analyzed, the target analytes, that is, GL and DGuA, could not be detected, while DGA exhibited a prominent peak. As a result, the simultaneous quantification of the target analytes and DGA, which was previously considered, was disregarded because the higher concentration of DGA would negatively impact the quantification of GL and DGuA. This observation was not considered a drawback as the goal and novelty of this study were to quantify GL and DGuA, simultaneously. The development of an external calibration curve for DGA, solely, using the methodology proposed in this study, should be considered as an alternative for the quantification of this compound. Furthermore, numerous methods have been validated and reported in the literature with the purpose of quantifying DGA [10, 11, 13].

At a following attempt, sample 1 fortified with a standard mix containing only GL and DGuA was analyzed, revealing that although GL and DGuA peaks could be detected, the resolution between DGA and DGuA was poor. To solve the coelution problem observed, the GC method was optimized and the original 30 meter DB-5MS capillary column was replaced by a 60 meter DB-624 column. The peaks identity was then confirmed by sample fortifications with GL and DGuA at 10, 25, and 100 ppm. The qualification of the optimized method was attempted by analyzing the unspiked sample 1, when it was observed that its sensitivity was still low for the detection of DGuA. It was assumed that, in order to develop sensitive calibration curves, the stock standards would need to be prepared with higher concentrations of DGuA. As described in Table 1, the stock standards were prepared with concentrations of DGuA three times higher than the concentrations of GL.

### 3.2. Method Qualification

#### 3.2.1. Specificity

**Figure 3:**
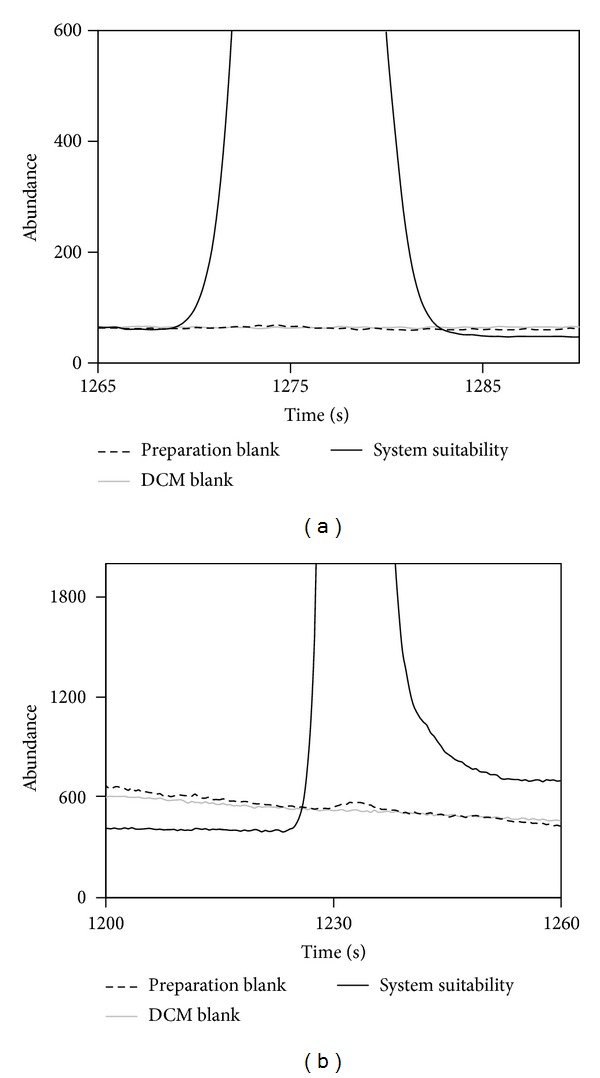
Specificity evaluation: extracted chromatograms of (a) ion 230 (GL) and (b) ion 217 (DGuA) of system suitability standard, solvent blank, and preparation blank.

Specificity is defined as the capability of the analytical procedure to identify the target analytes in the presence of other components that may be expected to be present. The specificity of the proposed method was evaluated by analyzing a system suitability standard at level 5, a solvent blank, and a preparation blank. The solvent and preparation blank were analyzed after the analysis of the system suitability standard to verify the presence of carry-over effect. As demonstrated in the extracted chromatograms of ions 230 (GL) and 217 (DGuA), displayed in Figure 3, no interfering peaks at the retention times of GL and DGuA were present.

#### 3.2.2. Precision

The precision of an analytical method describes the closeness of individual measures of an analyte when the procedure is applied repeatedly to multiple aliquots of a single matrix [20]. Precision was determined in this study by the relative standard deviance (RSD) of the peak area in repeated samples. Six injections of the system suitability solution in two different days were performed. The RSD for GL and DGuA obtained in the first and second days was, respectively, 9% and 2% and 8% and 6%. The RSD values obtained did not exceed the acceptance limit of 15% established by U.S. FDA [20]. In addition, the retention times of the analytes were very stable, with relative standard deviance (RSD) values of 0.01% for GL and 0.02% for DGuA.

#### 3.2.3. Linearity and Recovery

**Figure 4:**
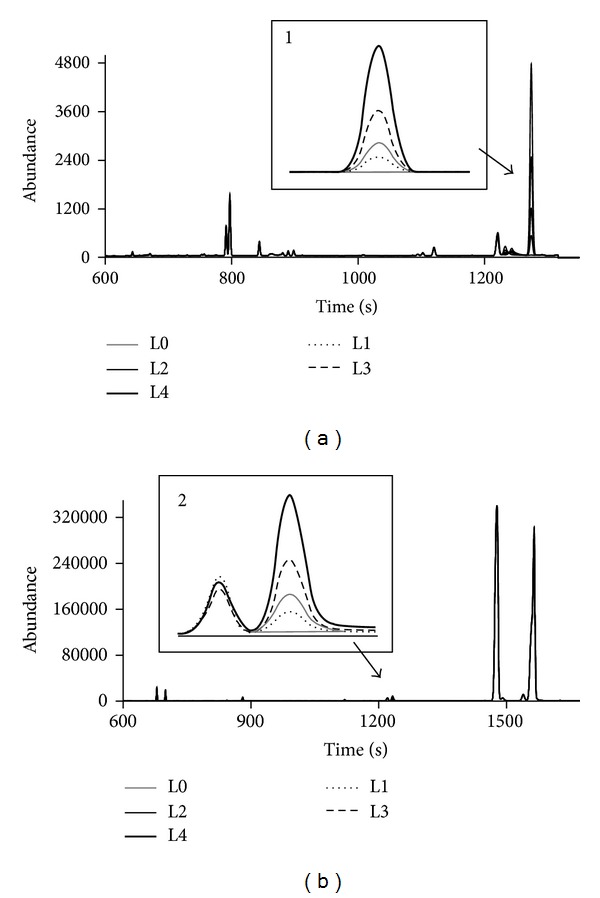
Representative SIM GC-MS ion chromatograms of (a) sample 2, ion 230 (229.7 to 230.7), and (b) sample 2, ion 217 (216.7 to 217.7).

**Figure 5:**
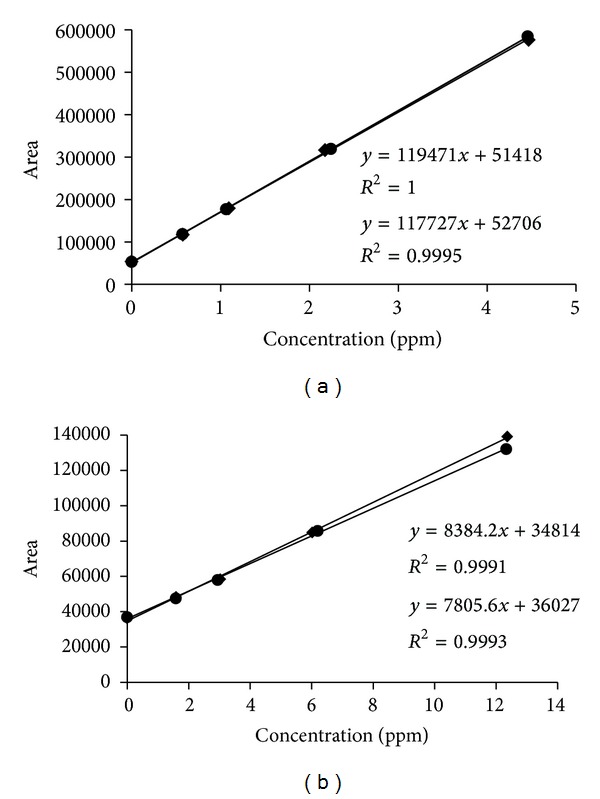
Calibration curves obtained in duplicate for (a) GL and (b) DGuA in sample 4 by CG-MS using the standard addition method.

Each of the four beverage samples was fortified with GL and DGuA at four levels of concentration, in duplicate, and submitted to the optimized method procedure to evaluate its ability to provide test results that are linearly proportional to the concentration of the analyte in the sample. The chromatograms obtained for sample 2 are shown in Figure 4. The GL and DGuA peaks can be clearly identified. Calibration curves for each of the beverage samples were generated to investigate the linearity of the detector response* versus* GL and DGuA concentrations. Figures 5(a) and 5(b) show the calibration curves obtained for sample 4, where it can be noticed that the GC-MS response linearly changed with the concentration of GL and DGuA added, respectively. As indicated in Table 2, excellent linearity results were observed for GL curves, with correlation coefficients ranging from 0.995 to 0.999. Lower correlation coefficients, ranging from 0.97 to 0.999, were observed for the DGuA calibration curves. These results were considered satisfactory as the analysis involved a derivatization step and a standard addition curve at low concentrations.

The percent recovery by the assay of known added amount of analyte in the samples was used to estimate the accuracy of the method. The recovery experiment was performed by comparing the calculated amount of the analytes with the real amount spiked to the samples.  Table 3 lists the average recovery yields of GL and DGuA in the beverage matrices at each level of concentration and its respective percentage of RSD. The average recovery yields ranged from 85 to 112% and were considered satisfactory. Most of the percentage values of recovery yields were obtained with acceptable variation (%RSD < 6), indicating that at these levels the recovery yields could be estimated with acceptable accuracy. Higher percentages of RSD, ranging from 14 to 28%, were observed at the lowest concentration levels of GL and DGuA.

#### 3.2.4. Limits of Detection (LOD) and Limits of Quantification (LOQ)

The LOD is defined as the lowest concentration of an analyte that an analytical procedure can reliably differentiate from background noise, while LOQ is defined as the lowest amount of an analyte in a sample that can be quantitatively determined with suitable precision and accuracy [20]. In this study, the LOD and LOQ calculated from the measurement of each sample were estimated considering signal-to-noise ratios of 3 and 10, respectively. One replicate of each sample was used to the calculation of the LOD and LOQ. The obtained results are reported in Table 4. A direct comparison of the results obtained by Suzuki et al. [16] in beverage samples and the ones obtained in this study is difficult to be accomplished, as in the first study the LOD and LOQ values were not presented. However, the working range in the study by Suzuki and coworkers was considerably higher. The authors reported that the calibration curves for GL and DGuA exhibited good linearity at least in the range from approximately 0.00317 mg to 0.10039 mg.

### 3.3. Quantification of GL and DGuA in Beverages

A standard addition method was chosen to quantify the actual content of GL and DGuA in the beverage samples. This decision was taken considering the high complexity of the sample matrices, the low levels of the analytes in the beverages, and the fact that there was no ideal blank sample available. The quantification results, obtained in duplicate, are shown in Table 5. The %RSD values for samples 1, 3, and 4 were considered satisfactory, ranging from 2% to 18% and indicating a good precision. A higher %RSD value, 28%, was observed on the quantification of GL in sample 2. A comparison of the calculated concentrations with the LOQ concentrations indicated that the estimated values could be determined with suitable accuracy, with the exception of the GL concentration in sample 2. Nonetheless, the concentration estimated (0.009 ppm) was found to be above the LOD and, therefore, to be accurately detected.

To our knowledge, the only previous study that aimed to quantify GL and DGuA in beverages was reported by Suzuki et al. [16]. One of the drawbacks of this HPLC-UV method is that it was only able to quantify the 1-phenyl-3-methyl-5-pyrazole derivatives of DGuA, exclusively. In the first step of the derivatization reaction, DGuA and GL were dissolved in an aqueous solution of sodium hydroxide. As DGuA, at basic conditions, is easily converted to its free-acid form (i.e., DGuA) by spontaneous hydrolysis of intramolecular ester linkage, both compounds were analyzed as the 1-phenyl-3-methyl-5-pyrazolone derivatives of DGuA. Indeed, the analysis of uronic acids by GC-MS encounters several analytical difficulties, especially related to the achievement of an efficient derivatization. In water solutions, monosaccharides undergo intramolecular reactions to form cyclic hemiacetals, hemiketals, and lactones which are in equilibrium with each other. To avoid the formation of multiple chromatographic peaks, which causes irreproducible quantification, loss of sensitivity, and unreliable identifications, some strategies have been described [21]. The conventional strategy compresses the formation of only one derivative from more than one sugar by reducing the carbonyl moieties to hydroxymethyl groups followed by acetylation [17]. In our study, BSTFA + 1% TMCS were successfully used for the derivatization of GL and DGuA under ultrasonic conditions, leading to the formation of one derivative to each compound.

## 4. Conclusion

In the present study a reliable GC-MS method for the quantification of GL and DGuA in beverages using the standard addition method was developed. The sample preparation involved a precolumn derivatization leading to the formation of one derivative to each compound. The choice of the capillary column was critical for the GC-MS analysis because of the low resolution between DGuA and DGA. The coelution problem was solved using a 60 meter DB-624 column and modifying the concentration of the calibration stock standards. The optimized method was sensitive enough to detect and quantify the analytes at low concentrations. For all beverage samples assayed, the validation figures of merit, namely, precision, specificity, linearity, recovery, and limits of detection and quantification, were considered satisfactory. In particular, LOD and LOQ values as low as 0.006 ppm and 0.02 ppm, respectively, were obtained.

A major disadvantage of the standard addition method is the necessity for constructing separate calibration curves for each sample, which can be time-consuming. However, the standard addition method provides high accuracy and was especially useful in this study since there was no ideal blank sample available. The proposed CG-MS method may represent a convenient and reliable alternative for the simultaneous investigation of GL and DGuA in the pharmaceutical and nutraceutical industries.

## Figures and Tables

**Table 1:** Calibration stock standards.

Standard level	Mass of mix stock (g)	Final mass (g)	Concentration (ppm)	
			GL	DGuA
SL1	NA	NA	0	0
SL2	0.117	10.019	1.3	3.58
SL3	0.229	10.592	2.39	6.62
SL4	0.518	10.037	5.71	15.81
SL5	1.006	10.027	11.11	30.75
SL6	2.011	10.072	22.12	61.19
SL7	4.033	10.001	44.62	123.47

**Table 2:** Slopes and calibration parameters of calibration curves for GL and DGuA.

Sample/replicate	GL				DGuA			
	Slope	*y*-intercept	*R* ^2^	Standard range (ppm)	Slope	*y*-intercept	*R* ^2^	Standard range (ppm)
1/1	364463	6770.7	0.997	0.11–1.10	20566	2357.0	0.97	0.31–3.05
1/2	348155	7874.9	0.995	0.14–1.10	19388	2502	0.98	0.41–3.05
2/1	199719	1910.9	0.998	0.13–1.10	8254.50	3002.0	0.996	0.36–3.08
2/2	201892	1174.1	0.998	0.13–1.06	8383.1	2363.9	0.995	0.36–2.92
3/1	273943	861850.0	0.995	0.57–4.41	2432	93900	0.998	1.58–12.20
3/2	288786	909809	0.999	0.58–4.31	2558	99701	0.992	1.60–11.94
4/1	117727	52707	0.999	0.58–4.47	8384.40	34811	0.999	1.59–12.36
4/2	119471	51417	0.999	0.57–4.46	7805.6	36027	0.999	1.58–12.33

**Table 3:** Average recovery yields (%) and relative standard deviation (%) at each level of concentration in spiked beverage samples (*n* = 4 at each level, i.e., 2 replicates and 2 injections).

Sample		GL				DGuA			
		Concentration level^1^				Concentration level^1^			
		1	2	3	4	1	2	3	4
1	% recovery	99.10	109.01	101.24	99.38	94.20	85.72	90.14	103.57
	% RSD	27.90	9.12	0.65	3.23	14.66	5.44	3.43	4.17

2	% recovery	85.16	111.86	100.18	99.63	95.42	111.43	100.47	99.37
	% RSD	1.99	3.29	1.88	1.57	15.10	4.77	6.83	2.44

3	% recovery	105.69	91.58	99.18	100.10	88.82	87.96	101.58	99.90
	% RSD	23.90	4.91	11.14	4.80	6.70	3.69	9.13	3.13

4	% recovery	96.58	98.57	101.49	99.79	97.60	94.20	100.78	100.18
	% RSD	2.96	1.15	1.63	1.75	6.64	1.94	2.16	4.02

^1^1 is the lowest and 4 is the highest level of concentration in spiked samples.

**Table 4:** Estimated limits of detection and quantification for GL and DGuA.

Sample	GL		DGuA	
	Sample LOD (ppm)	Sample LOQ (ppm)	Sample LOD (ppm)	Sample LOQ (ppm)
1	0.14	0.47	1.1	3.8
2	0.006	0.02	0.09	0.28
3	0.02	0.08	0.4	1.2
4	0.008	0.03	0.06	0.2

**Table 5:** Quantification of GL and DGuA in beverage samples using the standard addition method.

Sample	GL			DGuA		
	Concentration (ppm)	Average concentration (ppm)	% RSD	Concentration (ppm)	Average concentration (ppm)	% RSD
1	1.8	2.0	14%	11.4	12.0	7%
	2.2			12.6		

2	0.009	0.008^1^	28%	0.362	0.3	18%
	0.006			0.28		

3	2.8	2.9	2%	3.5	3.5	2%
	2.9			3.6		

4	1.3	1.2	11%	11.7	11.9	2.4%
	1.1			12.1		

^1^Average concentration lower than LOQ but higher than LOD.

## Abbreviations

BSTFA + 1% TMCS: N,O-Bis(trimethylsilyl)trifluoroacetamide with 1% trimethylchlorosilane
DCM: Dichloromethane
DGA: D-Glucaric acid
DGuA: D-Glucuronic acid
GL: Glucuronolactone
DMF: Dimethilformamide
GC: Gas chromatography
HPLC: High-performance liquid chromatography
LOD: Limit of detection
LOQ: Limit of quantification
MS: Mass spectroscopy
RSD: Relative standard deviance
TMS: Trimethylsilyl.
